# Supplementary material for: Study of Catalytic CO2 Absorption and Desorption with Tertiary Amine DEEA and 1DMA-2P with the Aid of Solid Acid and Solid Alkaline Chemicals
Source: Molecules. 2019 Mar 13;24(6):1009. doi: 10.3390/molecules24061009 (PMC6470649; doi:10.3390/molecules24061009)
Supplement: Supplementary file 1 [file molecules-24-01009-s001.pdf]

Support Information

1.0 The experimental apparatus for absorption and desorption

The experimental apparatus of CO_2 absorption was similar to other researchers.[1]

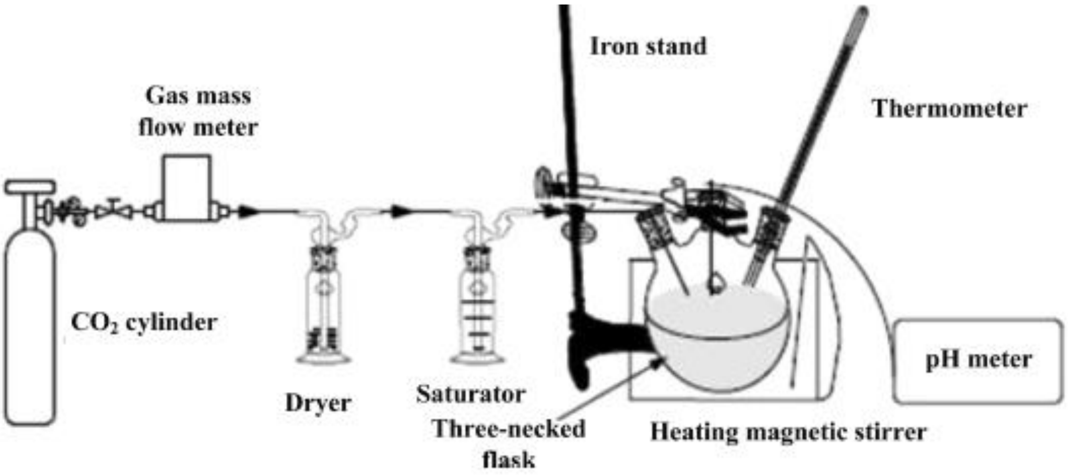

Fig. S1. Stirred Cell Reactor for CO_2-Amine interactions with a water scrubbing process.

Reference:

[1] Liu, H.; Idem, R.; Tontiwachwuthikul, P.; Liang, Z., Study of Ion Speciation of CO_2 Absorption into Aqueous 1-Dimethylamino-2-propanol Solution Using the NMR Technique. Industrial & Engineering Chemistry Research 2017, 56, (30), 8697-8704.

The experimental apparatus of CO_2 desorption was similar to other researchers. [2]

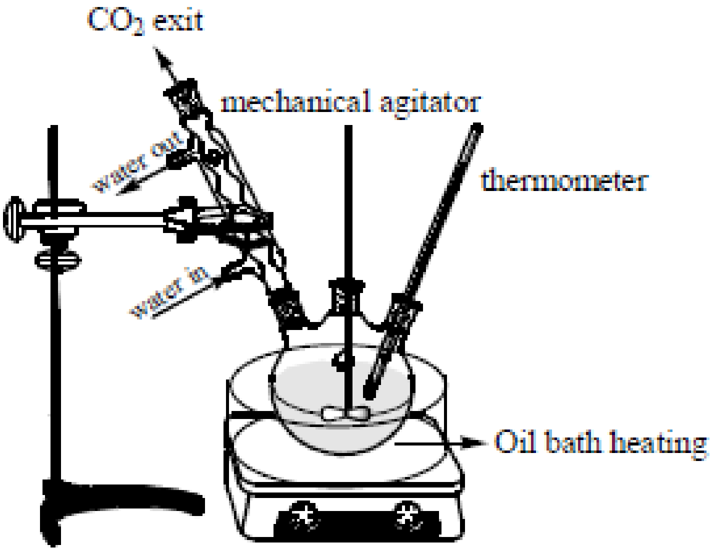

Figure S2. The schematic diagram of the CO_2 desorption process with oil bath. [2]

[2] Shi, H. C.; Zheng, L. N.; Huang, M.; Zuo, Y. H.; Kang, S. F.; Huang, Y. D.; Idem, R.; Tontiwachwuthikul, P., Catalytic-CO_2-Desorption Studies of DEA and DEA-MEA Blended Solutions with the Aid of Lewis and Bronsted Acids. Ind Eng Chem Res 2018, 57, (34), 11505-11516.

2.0 The CO_2 equilibrium solubility of several tertiary amines

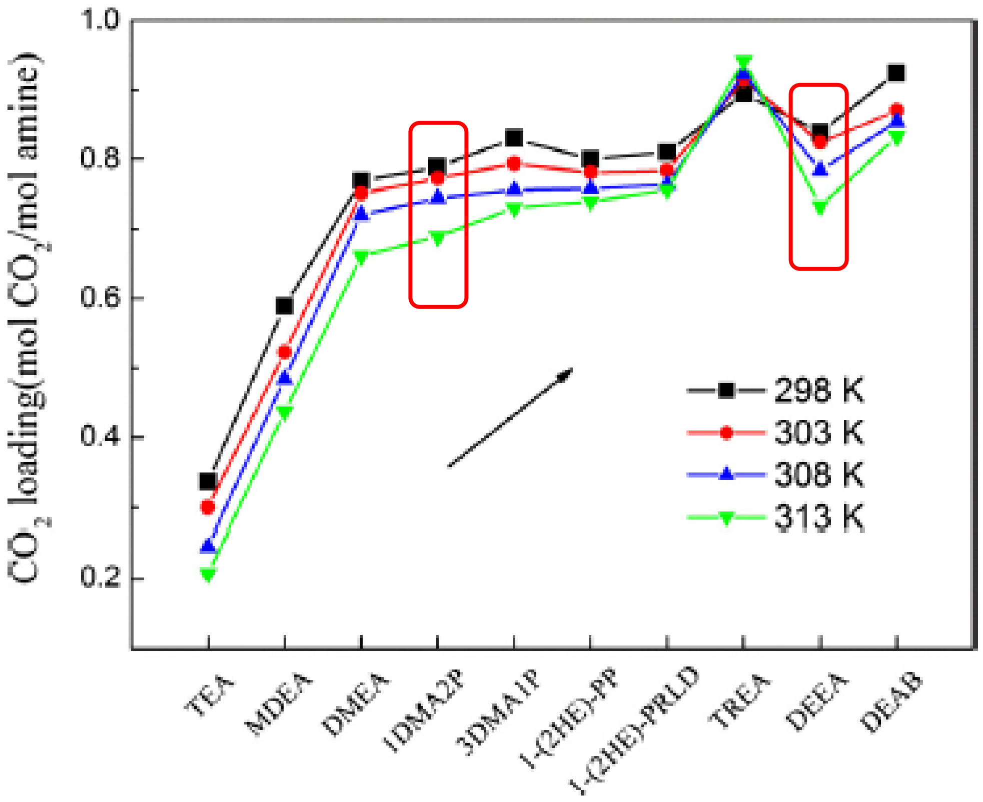

Figure S3. The CO_2 equilibrium solubility of investigated amines.[3]

(Fig 14 for reference [3])

Reference

[3] Min Xiao, H. L., Raphael Idem, Paitoon Tontiwachwuthikul, Zhiwu Liang, A study of structure–activity relationships of commercial tertiary amines for post-combustion CO_2 capture. Applied Energy 2016, 184, 219-229.

3.0 The order the solid acid catalysis for MEA and DEA as references

Table S1. The order of catalysis for MEA, DEA and MEA+DEA blended amines under different cases of blended solvents. [2]

T = 363K	Rich region 0.50-0.30 mol/mol	
5.0 DEA	H-ZSM-5 > γ-Al_2O_3 > blended catalyst	
0.5 MEA + 4.5 DEA	H-ZSM-5 > γ-Al_2O_3 > blended catalyst	similar to DEA
1.0 MEA + 4.0 DEA	H-ZSM-5 > γ-Al_2O_3 > blended catalyst	similar to DEA
1.5 MEA + 3.5 DEA	H-ZSM-5 ≈ γ-Al_2O_3 ≈ blended catalyst	Transition
2.0 MEA + 3.0 DEA	H-ZSM-5 > blended catalyst > γ-Al_2O_3	similar to MEA
2.5 MEA + 2.5 DEA	H-ZSM-5 > blended catalyst > γ-Al_2O_3	similar to MEA
MEA[5]	H-ZSM-5 > blended catalyst > γ-Al_2O_3	
**T = 378K**	**Lean region 0.30-0.15 mol/mol**	
5.0 DEA	**blended catalyst > H-ZSM-5 > γ-Al_2O_3**	
0.5 MEA + 4.5 DEA	**blended catalyst > H-ZSM-5 > γ-Al_2O_3**	similar to DEA
1.0 MEA + 4.0 DEA	blended catalyst > H-ZSM-5 > γ-Al_2O_3	similar to DEA
1.5 MEA + 3.5 DEA	blended catalyst > γ-Al_2O_3 > H-ZSM-5	similar to MEA
2.0 MEA + 3.0 DEA	blended catalyst > γ-Al_2O_3 > H-ZSM-5	similar to MEA
2.5 MEA + 2.5 DEA	blended catalyst > γ-Al_2O_3 > H-ZSM-5	similar to MEA
MEA[4]	blended catalyst > γ-Al_2O_3 > H-ZSM-5	

Reference

[2] Shi, H. C.; Zheng, L. N.; Huang, M.; Zuo, Y. H.; Kang, S. F.; Huang, Y. D.; Idem, R.; Tontiwachwuthikul, P., Catalytic-CO_2-Desorption Studies of DEA and DEA-MEA Blended Solutions with the Aid of Lewis and Bronsted Acids. Ind Eng Chem Res 2018, 57, (34), 11505-11516.

[4] Liang, Z. W.; Idem, R.; Tontiwachwuthikul, P.; Yu, F. H.; Liu, H. L.; Rongwong, W., Experimental study on the solvent regeneration of a CO2-loaded MEA solution using single and hybrid solid acid catalysts. Aiche J 2016, 62, (3), 753-765.
